# Phytochemical Diversity, Extraction Methods, Analytical Profiling, and Quality Control of *Rheum tanguticum* (Maxim. ex Regel) Balf.: An Evidence-Stratified Narrative Review

**DOI:** 10.3390/molecules31213874

**Published:** 2026-10-27

**Authors:** Mahmoud Soliman, Cheng Du, Linli Cheng

**Affiliations:** 1 College of Veterinary Medicine, China Agricultural University, Beijing 100084, Chinab20263050716@cau.edu.cn (C.D.); 2 Department of Animal Production, Faculty of Agriculture, Kafrelsheikh University, Kafr El Sheikh 35111, Egypt

**Keywords:** *Rheum tanguticum*, anthraquinones, stilbenes, extraction, LC–MS/MS, spatial metabolomics, authentication, quality control, evidence stratification

## Abstract

*Rheum tanguticum* (Maxim. ex Regel) Balf. is one of three botanical sources of Dahuang and exhibits chemical and processing variability that complicates species attribution and quality assessment. This review aims to synthesize species-specific phytochemical, extraction, analytical, pharmacological, and quality-control evidence and to propose a research framework tailored to the product matrix. A targeted narrative search of PubMed, Europe PMC, DOI-linked publisher records, and official botanical and regulatory sources covered material available through 18 September 2026; evidence was classified as authenticated direct studies (A), species-derived isolates (B), comparative *Rheum* evidence (C), or the broader compound-level literature (D), with authentication uncertainty stated explicitly. The synthesis shows that reported extraction outcomes are not directly comparable: crude decoction yield, target-analyte recovery, and chromatographic purity measure different endpoints. The current Chinese Pharmacopeia includes a Dahuang monograph, but its drug-level status should not be mistaken for a validated *R. tanguticum*-specific chemical profile. Five free anthraquinones are analytically accessible compositional markers but are not species-specific identifiers; glycosides, sennosides, stilbenes, tannin-related chemistry, phenylbutanones, and polysaccharides require matrix- and evidence-specific interpretation. We conclude that no single marker establishes botanical identity, efficacy, or safety. A defensible research strategy combines official compliance with voucher-supported authentication, validated multi-class profiling, processing-sensitive measurements, and safety assessment. The proposed panels are research priorities, not validated release specifications.

## 1. Introduction

### 1.1. Botanical Identity and Ecological Context

*Rheum tanguticum* (Maxim. ex Regel) Balf. is a perennial member of Polygonaceae associated with the Qinghai–Tibetan Plateau and adjacent regions. Flora of China describes deeply divided leaves and stout yellow roots and rhizomes and records the species in valleys at 1600–3000 m in Gansu, Qinghai, Shaanxi and Xizang. This floristic range should not be conflated with the elevation envelope of every cultivated or experimentally sampled population [1]. The dried roots and rhizomes contribute to Dahuang alongside *R. palmatum* and *R. officinale*; the shared drug name does not establish chemical equivalence among these botanical sources [2].

Population-marker studies of *R. palmatum* and *R. tanguticum* indicate close genetic relationships, while genetically identified material can show distinguishable chemical profiles [3,4]. Therefore, the species designation reported in a study should be accompanied, wherever available, by voucher information, collection locality and organ identity. Roots and rhizomes are different organs and should not be used as interchangeable sample descriptions.

Organ-level chemical analyses and root imaging support spatial heterogeneity in *R. tanguticum* [5,6]. These observations justify recording the sampled organ and tissue fraction in quality studies; they do not by themselves establish an optimal harvest age, a biosynthetic location or a universal ranking of medicinal quality.

### 1.2. Historical Significance and Pharmacological Context

The ethnomedical history of rhubarb concerns several botanical sources rather than a continuously authenticated single species [7]. Historical research on Japanese rhubarb further shows that product names and botanical origins changed over time [8]. Accordingly, historical duration-of-use claims should be assigned to rhubarb preparations, not automatically to modern taxonomically defined *R. tanguticum*.

The European Medicines Agency recognizes specified preparations of *R. palmatum* and *R. officinale*, or their hybrids, for short-term treatment of occasional constipation. Its monograph scope should not be presented as a species-specific authorization for *R. tanguticum* [9]. Direct experimental evidence for *R. tanguticum* includes polysaccharide studies in chemically induced rodent colitis [10,11], antimicrobial testing against plant pathogens [12], and a study of a 60% ethanol-eluted fraction in high-fat-diet-fed mice [13]. These results concern particular preparations and experimental endpoints, not established clinical efficacy of the whole plant. Network-predicted targets in the anti-obesity study remain hypotheses unless independently tested.

### 1.3. Modern Quality Challenges

Chemical variability arises across botanical sources, populations, organs and processing states. Species-specific evidence includes altitude-associated metabolomic differences [14], differences among morphological variation types within *R. tanguticum* [15], and geographical variation in constituent profiles [16]. Environmental studies involving more than one rhubarb species cannot separate genotype, soil, climate and cultivation effects without suitable sampling and statistical control [17].

A nationwide evaluative inspection reported 152 compliant and 22 noncompliant batches among 174 rhubarb medicinal-material and decoction-piece samples, an approximately 87% pass rate [18]. This is a commercial-rhubarb surveillance result, not a prevalence estimate restricted to authenticated *R. tanguticum*.

Quality evaluation must distinguish botanical identity, composition, contaminant control and intended performance. Passing a chemical assay does not independently authenticate a species; conversely, correct species identity does not guarantee consistent constituent concentrations. Multi-component studies of rhubarb demonstrate the feasibility of broader chemical assessment, but proposed biological correlates require validation in the intended product and use context [19].

### 1.4. Scope, Novelty, Audience and Literature Search

This review is intended for pharmacognosists, natural-products chemists, analytical scientists, traditional-medicine researchers, and quality or regulatory specialists working on botanical authentication and product standardization [2,20]. Its novelty is an evidence-stratified synthesis that connects botanical provenance to constituents, extraction and processing, analytical confidence, pharmacological interpretation, and quality-control decisions, while separating current official requirements from research proposals.

The literature search was designed as targeted narrative coverage, not as a systematic review. PubMed and Europe PMC were searched from database inception through 18 September 2026. DOI-linked publisher records and Crossref-style bibliographic records were used to verify citations and retrieve accessible articles; Flora of China and official pharmacopoeial and regulatory resources were consulted separately. The regulatory status of the Chinese Pharmacopeia was rechecked against the National Medical Products Administration notice on 14 October 2026.

Representative search string (syntax adapted to each resource): (“*Rheum tanguticum*” OR “Rhei Radix et Rhizoma” OR Dahuang OR rhubarb) AND (phytochemistry OR anthraquinone* OR sennoside* OR stilbene* OR tannin* OR polysaccharide* OR metabolom* OR extraction OR decoction OR ultrasound OR HPLC OR LC-MS OR authentication OR “quality control” OR pharmacolog*). Focused searches paired *Rheum tanguticum* with extraction-method terms (heat reflux, decoction, ultrasound-assisted extraction, counter-current chromatography, yield), analytical terms (HPLC, LC-MS/MS, QAMS, NIR, MALDI-MSI, validation), and pharmacopeia or regulatory terms.

Eligible sources included primary studies on *R. tanguticum* or compounds explicitly reported as isolated from it; comparative or other-species *Rheum* studies when used only for context; broader compound-level studies when relevant to mechanistic or safety interpretation; and official botanical, pharmacopoeial, or regulatory documents. Reviews were used for context and reference checking, not as sole evidence for a species-level isolation or identification claim. Studies unrelated to *Rheum*, papers without interpretable chemical or botanical attribution, and unsupported repeated secondary claims were excluded from species-specific conclusions.

Titles and abstracts were screened for topical relevance, followed by full-text review when accessible and backward reference checking. Chinese-language studies were retained when accessible bibliographic records or abstracts supported interpretation; experimental details not available in the accessible record were not inferred. Overlapping records were manually reconciled by DOI, title, and author/year; no PRISMA flow, independent duplicate-count log, or formal dual-reviewer screening was maintained. The review therefore reports no pooled study count or meta-analysis. Evidence categories are provenance labels, not rankings of study quality: A, authenticated direct *R. tanguticum* material; B, isolates explicitly obtained from material reported as *R. tanguticum* (voucher authentication may remain unresolved); C, comparative or other-species *Rheum* evidence; and D, the broader compound-level or pharmacological literature. Where authentication was not independently verifiable, studies are designated species-reported rather than assigned to A.

## 2. Chemical Constituents

The chemical repertoire of *R. tanguticum* includes several small-molecule families and heterogeneous polysaccharide fractions. A fixed species-wide count is currently inappropriate without a deduplicated, structure-resolved inventory linked to authenticated specimens. Jin et al. reported 41 constituents identified or tentatively characterized in one liquid chromatography–diode-array detection–electrospray ionization mass spectrometry (LC-DAD-ESI-MS) investigation: 16 anthraquinone derivatives, seven phenylbutanone glucopyranosides, four stilbenes and 14 tannins [21]. Gao et al. tentatively identified 30 constituents using ultraperformance liquid chromatography/quadrupole time-of-flight mass spectrometry (UPLC/Q-TOF-MS), with seven reference standards informing interpretation [22]. These are study-specific analytical counts, not additive totals of newly isolated molecules.

**Figure 1:**
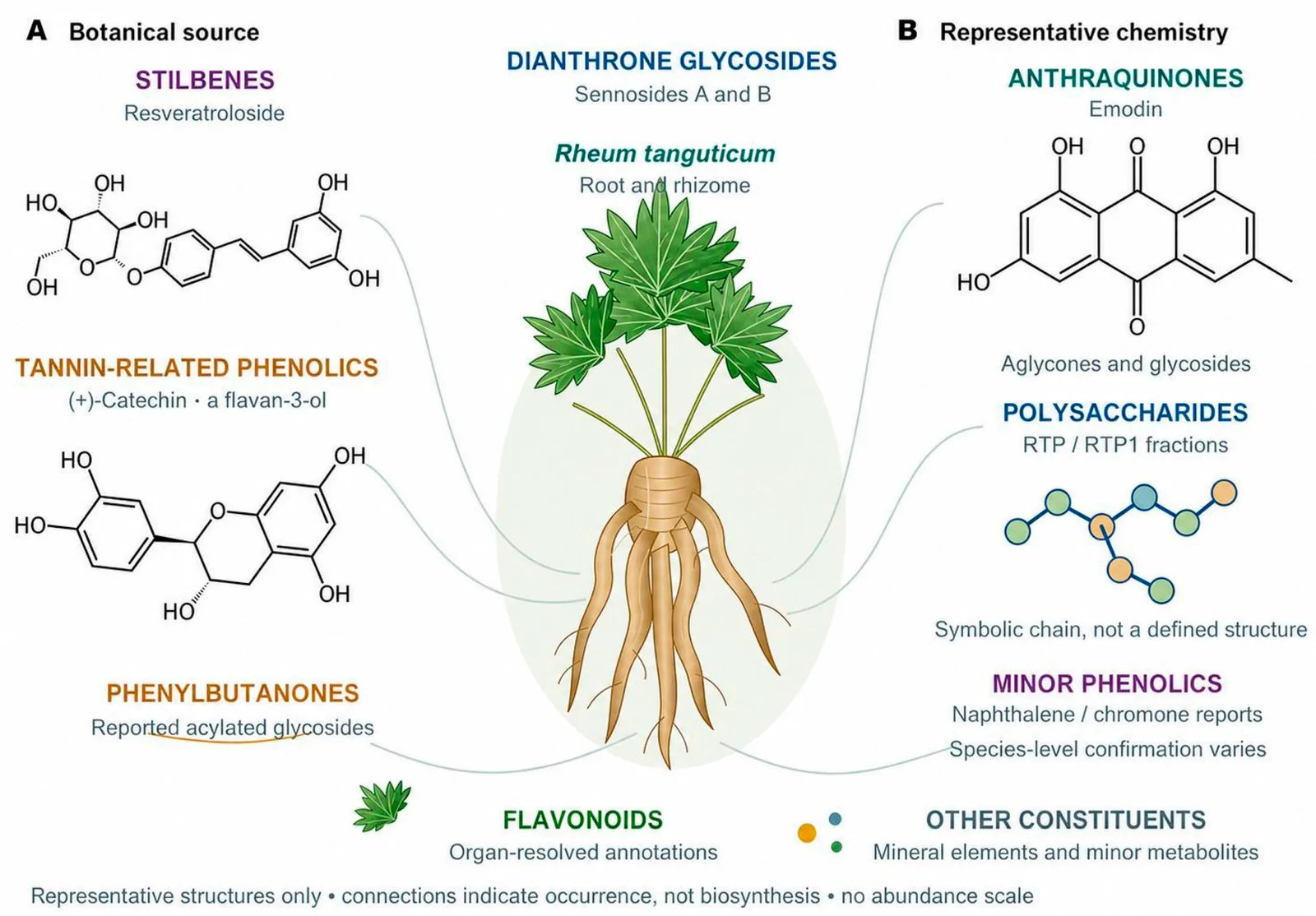
Representative phytochemical landscape of R. tanguticum. (**A**) Botanical source (the plant and root/rhizome are schematic, not diagnostic botanical illustrations); (**B**) Representative chemistry and chemical classes. Emodin, resveratroloside, and (+)-catechin were drawn from verified PubChem stereochemical records (CIDs 3220, 5322089, and 9064) [24]. Their reported isolation from R. tanguticum is supported by primary studies [25,26], but source attribution does not independently verify voucher authentication. Catechin is a flavan-3-ol, not a polymeric tannin. RTP/RTP1 identify study-defined polysaccharide preparations; the symbolic chain does not specify linkage or sequence. Chemical domains indicate reported occurrence, not abundance or biosynthetic relationships. Minor-constituent and source-authentication limitations are discussed in the text [1,10,11,15,22,23,27].

More recent high-coverage metabolomics reports substantially larger annotated metabolite sets, but these do not constitute equivalent numbers of purified, structurally established natural products [23]. Figure 1 summarizes the chemical landscape without encoding abundance, class percentages or unsupported inventory totals. Table 1 distinguishes evidence types and functional interpretation.

### 2.1. Anthraquinone Derivatives

Free and glycosylated anthraquinones are central to chemical profiling, but “total anthraquinones” is an operational analytical endpoint. A result obtained after hydrolysis is not directly interchangeable with the sum of directly measured native aglycones and glycosides. Results should specify extraction, hydrolysis, calibration, moisture correction and reporting basis [33,37].

#### 2.1.1. Free Anthraquinones

Emodin, rhein, chrysophanol, aloe-emodin and physcion are recurring analytical targets [21,22]. Table 2 gives their neutral molecular formulas and average molar masses; these are not exact masses or the *m*/*z* values of deprotonated ions. The shared formulas of emodin and aloe-emodin illustrate why nominal or accurate mass alone cannot resolve constitutional isomers.

Formulas in Table 2 are chemical descriptors, not concentration data. Molar masses are reported database values for the stated neutral formulas, cross-checked against PubChem [24]. Compound occurrence is supported by [21,22]; pharmacological evidence is evaluated separately in Section 5.

Reviews of emodin describe multiple preclinical activities together with poor exposure, toxicity concerns and translational limitations [38,39]. The compound-level literature does not establish the efficacy or safety of a chemically variable *R. tanguticum* extract. Nor does successful validation of an assay demonstrate clinical benefit.

Genome assembly and transcriptomics provide a basis for studying anthraquinone biosynthesis in *R. tanguticum* [40,41,42]. These datasets nominate candidate enzymes, whereas gene expression correlations do not establish catalytic function. Reference-gene validation supports more reliable expression measurements under specified stress conditions but is not itself a biosynthetic-pathway experiment [43]. A 2026 multi-omics study added transformation-based evidence concerning RtPKSIII-8 and RtUGT85AD11, strengthening the direct species evidence without resolving every biochemical step [23]. Alternative-splicing analyses of the *R. palmatum* complex remain comparative evidence whose taxon and accession assignments must be preserved [44].

#### 2.1.2. Conjugated Anthraquinones

Preparative studies provide firmer structural support than an undifferentiated list of LC-MS features. Resin enrichment followed by HSCCC separated aloe-emodin 8-O-β-D-glucoside, emodin 1-O-β-D-glucoside and emodin 8-O-β-D-glucoside from *R. tanguticum* [28]. Preparative HPLC combined with HSCCC also addressed the difficult separation of chrysophanol 1-O- and 8-O-glucosides and a physcion glucoside [29]. Table 3 presents a supported representative list rather than a complete species inventory.

Glycosylation changes physicochemical properties and may alter absorption, microbial metabolism and assay responses. It is not defensible to classify every glycoside as an inactive prodrug or to assume that deglycosylation always increases activity. In *R. officinale*, characterized UGTs catalyze emodin glycosylation; this is direct evidence for that species and comparative evidence for *R. tanguticum* [45]. Structural numbering also requires consistency: alternative orientation of the anthraquinone scaffold can produce apparently different locants. An “emodin 6-glucoside” cannot be accepted or rejected solely by comparing its name with a differently numbered aglycone. Structure drawings, atom numbering and spectroscopy must resolve the assignment.

### 2.2. Anthrone Derivatives and Sennosides

Anthrones, dianthrones and dianthrone glycosides should be distinguished chemically. Sennosides A and B are dianthrone glycosides, not free anthraquinones. Their determination in *R. tanguticum* has been reported in variation-type and multi-class constituent studies [15,27]. Table 4 summarizes supported analytes without inferring a complete species inventory.

The established metabolic rationale for sennosides includes microbial transformations that generate rheinanthrone; hydrolysis alone is an incomplete account because reduction is also involved [30]. In vitro fermentation of *R. officinale* extracts provides related evidence but cannot determine exposure or efficacy in humans taking *R. tanguticum* [46]. “Pharmacopoeial marker” is edition-, jurisdiction- and monograph-specific; sennosides A and B are not labeled as official Chinese Dahuang assay markers (see Section 6.1).

### 2.3. Stilbenes and Stilbene Glycosides

The species-specific preparative literature includes trans-3,5,4′-trihydroxystilbene-4′-Oβ-D-glucopyranoside, commonly termed resveratroloside, isolated with (+)-catechin [25]. It also includes trans-rhapontin, cis-rhapontin and trans-desoxyrhaponticin [31], and piceatannol 4′-O-β-D-(6″-O-gallate)-glucoside [28]. These reports predate 2018 and must not be described as 2018–2026 discoveries. Table 5 summarizes representative reported stilbenes and their authentication cautions.

Classical isolation work on commercial Chinese rhubarb characterized piceatannol 4′-O-β-D-glucopyranoside and its 6″-O-gallate [47]. Because the accessible record identifies the commercial material rather than an authenticated *R. tanguticum* voucher, this is retained as foundational rhubarb chemistry, not direct species attribution.

An unresolved conflict is important for authentication: one preparative study reports rhapontin-related compounds from material named *R. tanguticum*, whereas a comparative identification study reports no rhaponticin in its authentic rhubarb samples [31,32]. Different sample identity, provenance, sensitivity, nomenclature or analytical assignments could contribute; none is established as the explanation here. These compounds should not be treated as universal presence/absence markers until the underlying vouchers, chromatograms and structures have been reconciled. Transcript evidence or a stilbene synthase from another species cannot resolve this conflict or prove a compound’s occurrence in *R. tanguticum*.

### 2.4. Tannins

The tannin-related chemical space includes hydrolysable tannins, condensed tannins and associated low-molecular-weight phenolics. Gallic acid is a phenolic acid, while catechin and epicatechin are flavan-3-ols; their measurement is informative but does not directly quantify intact hydrolysable tannins or condensed tannin polymers. Multi-class LC-MS profiling and preparative isolation support the presence of tannin-related constituents in *R. tanguticum* [21,25]. Comparative quantitative methods covering galloylglucoses, procyanidins and related classes offer broader coverage [4,33].

Colorimetric totals, chromatographic sums and gravimetric fractions measure different endpoints. Their calibration equivalents, extraction efficiency and interference must be stated before comparisons are made. Antioxidant responses in chemical assays should not be translated directly into systemic antioxidant or haemostatic efficacy.

### 2.5. Polysaccharides

*R. tanguticum* polysaccharides are operationally isolated fractions rather than a single molecular entity. RTP1 was investigated in TNBS-induced colitis in rats, including oral administration at 200 mg kg^−1^ in the reported experimental design [10]. Another study characterized an acidic polysaccharide preparation and evaluated it in DSS-induced colitis in mice [11]. An intestinal epithelial-cell study used pretreatment with 30, 100 or 300 μg mL^−1^ RTP before hydrogen-peroxide challenge [48]. These are preparation- and model-specific findings, not evidence that all *R. tanguticum* polysaccharides share equivalent activity.

Fecal fermentation experiments additionally support interactions between an *R. tanguticum* polysaccharide preparation and microbial communities [34]. Fermentation outcomes cannot be equated with in vivo clinical benefit. Structural interpretation requires molecular-weight distribution, monosaccharide composition, linkage analysis, branching, uronic-acid content and purity assessment. Protein, phenolic and endotoxin carryover should be addressed in mechanistic studies. Fraction names are study-specific and should not be treated as structural synonyms without direct characterization.

### 2.6. Phenylbutanones and Chromones

Phenylbutanone glucopyranosides are supported by species-specific LC-MS profiling [21]. Gao et al. reported isolation of a new cis-p-coumaroylated phenylbutanone glucopyranoside together with 12 known compounds from *R. tanguticum* roots [35]. The 12 known co-isolates should not be interpreted as 12 additional phenylbutanones. Lindleyin and related compounds belong within a structure-resolved phenylbutanone inventory only when their individual assignments are supported.

Chromones remain less well substantiated in the accessible species-specific evidence than anthraquinones and selected stilbenes. General rhubarb reviews provide contextual leads [2], but an exact *R. tanguticum* chromone count or a chromone–anthraquinone synergy claim is not justified by the evidence assessed here. Isolation records and diagnostic spectral evidence are priority gaps.

### 2.7. Naphthalene Derivatives

Naphthalene-related constituents form a chemically distinct category and should not be combined automatically with anthraquinones. Species-specific high-resolution profiling contributes annotations of minor constituents [22], while the general rhubarb literature offers additional candidate names [2]. Exact substitution patterns require compound-level primary isolation evidence; no complete species inventory is established. Putative oxalyl, galloyl or methoxy substitutions require adequate mass-fragmentation and preferably NMR evidence; an unresolved feature should retain an annotation-level description.

### 2.8. Flavonoids

Organ-resolved metabolomics reports substantial flavonoid representation in *R. tanguticum*, particularly in leaves [23]. Seed analyses across ten lines further demonstrate that aerial reproductive tissues have their own bioactive-compound and antioxidant profiles [36]. These findings do not establish that root material contains the same compounds at comparable concentrations. Transcriptomic work on green and purple tissues supplies additional context for pigmentation and tissue differences, not a substitute for metabolite identification [49].

The altitude-gradient study reported annotations including luteolin, isovitexin, vitexin, myricetin and luteolin-7-O-glucuronide in root comparisons [14]. These are metabolomic findings, not a claim that all were newly isolated from the species.

Chemical nomenclature must distinguish O- from C-glycosides; for example, an apigenin 8-C-glycoside should not be represented as an 8-O-glycoside. Novel occurrence in a species, first tentative annotation and discovery of a new molecular structure are separate claims.

### 2.9. Volatile Compounds and Other Constituents

Mineral elements and other primary or specialized metabolites contribute to organ-level chemical differences [5,23]. Long-chain fatty acids should not collectively be labeled a volatile fraction without a defined analytical method and sample-preparation context. Likewise, apparent alkaloid annotations in untargeted datasets do not establish the unusual occurrence of matrine or oxymatrine. Such assignments require authentic standards, orthogonal spectra and exclusion of contamination, substitution and database misannotation. These unusual names are therefore excluded from the confirmed inventory rather than used to increase chemical diversity.

## 3. Extraction Methods

### 3.1. Overview and Comparison

**Figure 2:**
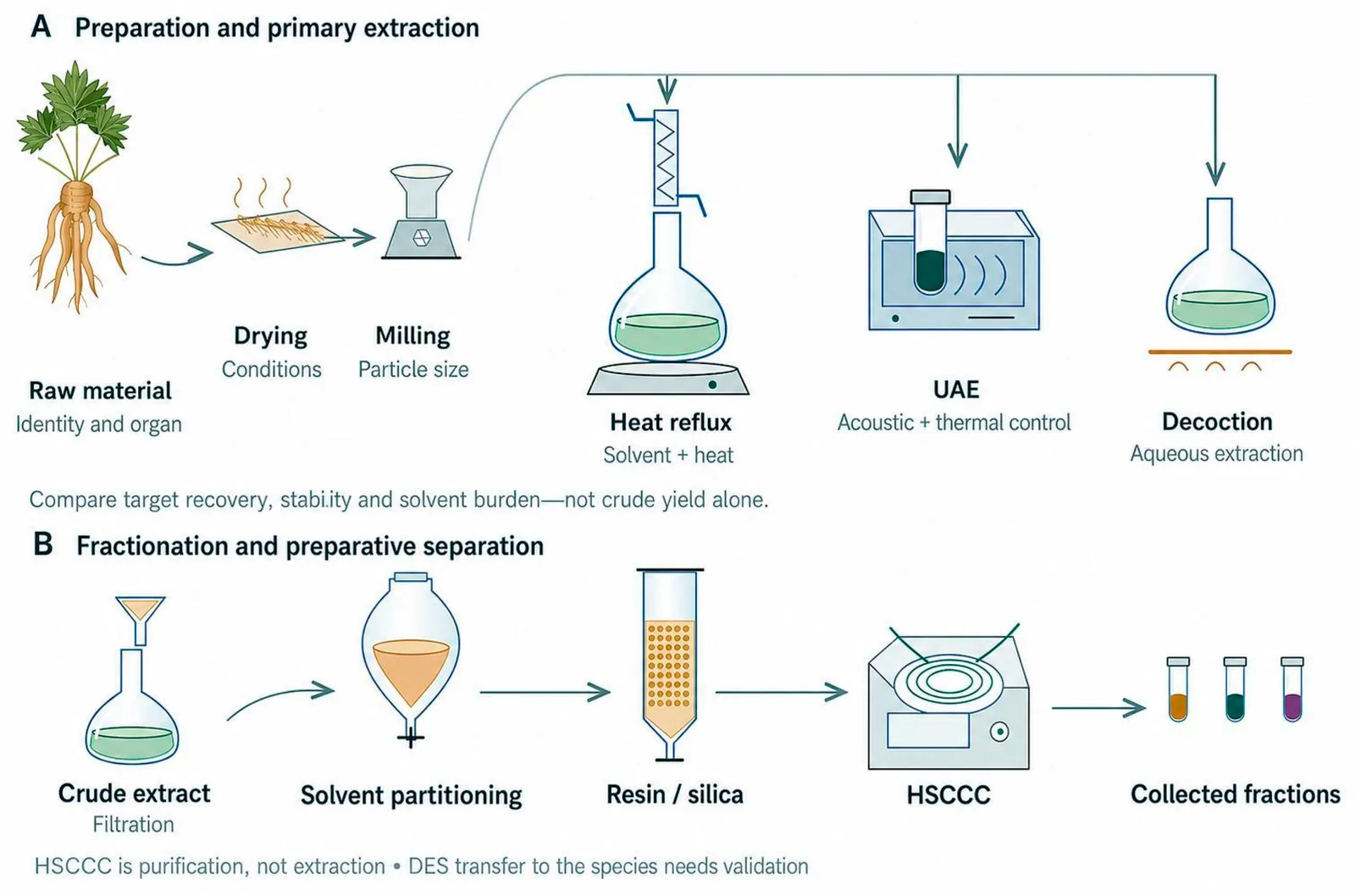
Illustrated preparation, extraction and preparative separation. (**A**) Drying and milling precede alternative thermal extraction and ultrasound-assisted extraction (UAE) approaches. (**B**) Filtration, solvent partitioning, adsorptive chromatography, and high-speed counter-current chromatography (HSCCC) illustrate available fractionation operations, not a single experimentally validated serial protocol. Arrows indicate possible handling routes, not reaction mechanisms or a performance ranking. Reported examples involve different organs, analytes, and designs; no universal extraction recipe or method ranking is implied [12,28,31,50,51,52,53,54,55,56]. DES, deep eutectic solvent. The comparative criteria and integrated organization are proposed by this review.

Primary extraction transfers constituents from plant material into a solvent; fractionation and chromatography enrich or purify the resulting extract. Heat reflux extraction (HRE), decoction and ultrasound-assisted extraction (UAE) are extraction approaches. High-speed counter-current chromatography (HSCCC) and silica/resin chromatography are downstream separation operations, although they can form part of an integrated preparation workflow. Figure 2 distinguishes these stages and the evidence-supported versus proposed components.

A comparative study of water- and ethanol-extracted *R. tanguticum* identified 24 differential metabolites and investigated circulating parent compounds and metabolites in rats, directly demonstrating that extraction choice changes the chemical material being assessed [50]. Crude extract yield, target-analyte recovery, chromatographic purity and biological response must therefore remain separate endpoints. Comparisons across unrelated studies are not head-to-head evidence of superiority.

Before extraction, botanical identity, root/rhizome selection, drying, milling and storage should be documented. A 2026 study found that rankings of drying methods depended on whether physical properties, free anthraquinones or broader metabolomic preservation were prioritized [51]. This supports endpoint-dependent pretreatment selection rather than a universally optimal drying method.

### 3.2. Heat Reflux Extraction (HRE)

#### 3.2.1. Solvent Screening

HRE offers reproducible solvent contact at a controlled thermal regime and is compatible with established laboratory equipment. Solvent polarity must be matched to the intended aglycone, glycoside or macromolecular fraction. Water can co-extract sugars and other matrix constituents, while aqueous alcohol changes selectivity; a larger residue mass does not necessarily mean greater recovery of relevant anthraquinones [50]. Methanol may be appropriate for analytical extraction or research isolation, but solvent acceptability and removal require separate consideration for medicinal manufacturing.

A species-specific analytical preparation used 0.15 g material with 25 mL methanol, refluxed for 1 h at a reported 65 °C, followed by solvent-loss correction. A 5 mL aliquot was evaporated, treated with 10 mL 8% hydrochloric acid, sonicated for 2 min, combined with 10 mL chloroform and refluxed for a further hour before chloroform partitioning and reconstitution [14]. This is a hydrolysis-containing analytical assay preparation, not an optimized manufacturing process or a measurement of native free anthraquinones alone.

Solvent rankings require matched material, an explicitly defined response and independently confirmed extraction conditions. Results obtained with different organs, preparation histories or quantification bases should not be combined into a universal solvent hierarchy.

#### 3.2.2. Parameter Optimization

A suitable optimization design separates temperature, time, solvent composition, solid-to-liquid ratio, particle size and number of extraction cycles. The tested ranges, fixed conditions and units must accompany any optimum. The recommended response is a justified set of target recoveries and stability measures, not residue yield alone. Model adequacy requires residual diagnostics, lack-of-fit assessment and replicated confirmation at the proposed operating point; an R^2^ value alone cannot demonstrate predictivity. Hybrid RSM–ANN–GA optimization has been reported for a rhubarb DES process, but botanical transfer to *R. tanguticum* requires verification [54].

### 3.3. Decoction Extraction

Decoction has direct relevance to aqueous preparations, but soluble macromolecules and small-molecule phenolics need different analytical endpoints. Water-extracted and ethanol-extracted *R. tanguticum* show different profiles [50]. Studies of stem material cannot establish optimum conditions for medicinal root or rhizome because their matrices differ. In a stem-specific study, an L9(3^4^) orthogonal design evaluated extraction frequency, duration and liquid-to-solid ratio, with crude yield, polysaccharides and malic acid as responses. The reported optimum was 12-fold distilled water, three decoctions of 1 h each; the abstract reports a study-defined mean crude extract yield of 38.65% [52]. The full methods are needed to reconcile the response denominator and composition bases before comparison with root preparations.

Prolonged heating may change native composition; distinguishing extraction from transformation requires time-resolved parent/product measurement. Species-specific scorch-processing work demonstrates that severe thermal treatment can lower anthraquinone glucosides while increasing aglycones [56], but its 180 °C treatment is not equivalent to boiling-water decoction. It therefore supports concern about processing-induced conversion, not an assumed decoction degradation rate.

### 3.4. Ultrasound-Assisted Extraction (UAE)

UAE should be evaluated through analyte recovery and integrity under defined sonication conditions. Bath or probe configuration, frequency, delivered power or energy, vessel geometry, actual sample temperature, treatment time and duty cycle are necessary for reproducibility. Particle size and solvent loading affect mass transfer and practical handling. These are proposed reporting requirements, not species-specific optima.

Direct species-specific evidence is available for chrysophanol 1-O-β-D-glucoside and chrysophanol 8-O-β-D-glucoside: response-surface optimization reported 41 min, 23 mL g^−1^ liquid-to-solid ratio and 40 °C [53]. The accompanying HPLC method used an Agilent Eclipse-XDB C18 column (250 × 4.6 mm, 5 μm, Agilent Technologies, Santa Clara, CA, USA), methanol–water gradient elution, 1.0 mL min^−1^ and 280 nm detection. Solvent strength, ultrasound energy and particle size are not specified in the accessible abstract and should not be supplied from assumption. This optimum concerns two chrysophanol glucosides, not sennoside A.

A separate *R. tanguticum* analytical study extracted 0.5 g samples with 25 mL 60% methanol for 1 h at 40 °C using reported settings of 240 W and 40 kHz to determine sennosides and tannin-related monomers [14]. These are reported analytical preparation conditions, not a demonstrated optimum or proof that one hour is equally suitable for every target. Together with the 41 min chrysophanol-glucoside protocol, they show why a single universal UAE time should not be inferred.

A lower measured concentration after longer extraction would not, alone, establish glycosidic-bond cleavage or an ultrasound-specific mechanism. Time-dependent changes require an analyte-specific mass balance and appropriate thermal controls; no universal UAE time is established.

### 3.5. High-Speed Counter-Current Chromatography (HSCCC) and Preparative Isolation

HSCCC is liquid–liquid chromatography without a solid stationary adsorbent. Zhao et al. separated three reported stilbene glycosides under the conditions in Table 6 [31]. Importantly, the products were trans-rhapontin, cis-rhapontin and trans-desoxyrhaponticin, not piceid, resveratroloside and rhaponticin. The reported loaded sample was 80 mg. The upper and lower phases should retain those physical labels: describing the upper phase as “organic” in a chloroform-containing system without phase-composition evidence is unsafe.

The values describe one reported separation, not plant dry-weight concentrations, recovery from raw roots or demonstrated industrial performance. The botanical/marker inconsistency discussed in Section 2.3 remains relevant.

An earlier study used stepwise ethyl acetate–ethanol–water systems, 25:1:25 and 5:1:5, to isolate resveratroloside and (+)-catechin. From a 250 mg crude load, it obtained 10.2 mg and 26.7 mg, respectively, with reported purities above 96%; flow increased from 0.8 to 2.0 mL min^−1^ after 5 h [25]. This study, rather than [31], supports resveratroloside isolation. A later resin/HSCCC workflow selected X-5 resin, used 40% ethanol elution, and employed chloroform–ethyl acetate–methanol–water (8:1:6:5) for subsequent separation [28]. Its analyte recoveries after resin elution were 89.0%, 85.9%, 82.3% and 84.9% for the four named targets in Section 2.1.2 and Table 3; these are not crude extraction yields.

### 3.6. Multi-Step Chromatographic Isolation

Bioactivity-guided fractionation of *R. tanguticum* rhizomes yielded anthraquinones and stilbenes active against phytopathogenic organisms [12]. Flash silica chromatography also provides a species-specific preparative precedent [26], while combined preparative HPLC and HSCCC address positional glycoside isomers [29]. These approaches offer structural and assay material, but purification loss, adsorption, solvent consumption and throughput differ from preparation of a standardized whole extract. Detailed solvent sequences should be assessed against the intended product and confirmed in the primary experimental record before transfer.

Emerging DES extraction deserves separate, qualified consideration. A rhubarb study investigated a TBAB–polyol system using hybrid RSM–ANN–GA optimization [54]. Its accessible abstract does not establish *R. tanguticum* as the botanical material or resolve all solvent-composition bases, so it is treated as a comparative methodological precedent rather than a species-specific recipe. Low volatility alone does not establish environmental superiority: solvent toxicity, recovery, product compatibility, residual solvent and energy consumption require evaluation.

For practical comparison, HRE favors established equipment but carries thermal and solvent burdens; decoction reflects aqueous use but co-extracts matrix and can change composition; UAE is a process-intensification option whose reproducibility depends on energy and temperature control. HSCCC avoids solid-phase adsorption but requires phase-system selection and can use hazardous solvents. Multi-step isolation supplies purified compounds rather than a directly comparable bulk extract. Green-process claims require process-level evidence rather than designation of a solvent as “green”. These are methodological considerations, not an experimentally established ranking.

The available outcome data demonstrate why method rankings must be endpoint-specific: crude dried-extract yield, target-analyte recovery, and purified-compound mass are not interchangeable. In the comparison compiled by Zhang and Chi [55], the SWE result comes from their experiment on *R. tanguticum* roots, whereas reflux, UAE, MAE, and SFE values are drawn from separate literature reports and do not constitute a matched, same-lot experiment. The source itself gives two UAE values (1.755% in its comparison table and 1.775% in the discussion); both are flagged in Table 7 pending verification against the cited primary study. Table 7 labels denominators and evidence scope; missing or non-comparable yields are stated rather than inferred.

## 4. Analytical Methods

### 4.1. High-Performance Liquid Chromatography (HPLC)

HPLC-DAD can provide reproducible fingerprints and targeted quantitative assays when reference standards, separation selectivity and matrix-specific validation are adequate. A species-specific fingerprint study assigned 21 characteristic peaks using reference-compound retention and UV data [57]. Broader rhubarb methods quantified 30 constituents and demonstrated botanical-source-associated patterns in genetically identified samples [4,33]. These studies offer stronger foundations than assuming that a method validated in an unrelated multi-herb formulation transfers unchanged to *R. tanguticum*.

**Figure 3:**
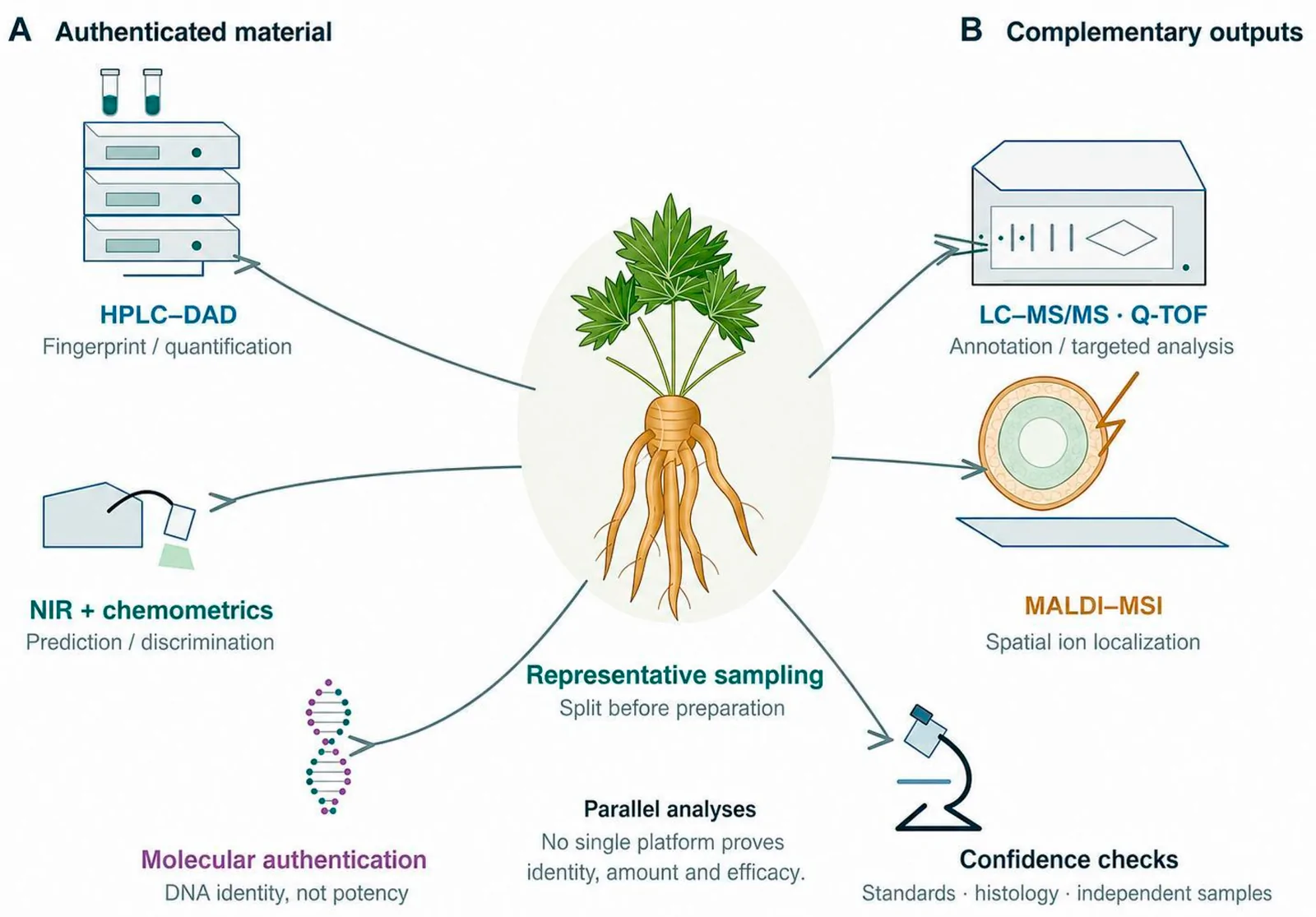
Complementary analytical and authentication approaches. (**A**) Authenticated material and representative sampling supplying parallel analyses rather than a compulsory serial platform sequence; (**B**) Complementary analytical outputs: HPLC–DAD supports fingerprinting and validated quantitative assays; LC–MS/MS and Q-TOF support confidence-graded structural assignments and targeted measurement. MALDI–MSI preserves tissue position, whereas NIR/chemometrics provides calibration-dependent prediction. Molecular authentication addresses botanical identity rather than chemical potency. Instrument and tissue symbols are conceptual; no spectra, chromatograms, ion maps, or classifier-performance data are displayed. The integration is proposed; applications of constituent methods are reported [6,21,22,23,33,37,57,58,59,60]. HPLC, high-performance liquid chromatography; DAD, diode-array detection; LC–MS/MS, liquid chromatography–tandem mass spectrometry; Q-TOF, quadrupole time-of-flight; MALDI–MSI, matrix-assisted laser desorption/ionization mass spectrometry imaging; NIR, near-infrared.

Table 8 compares analytical capabilities and validation limits. Figure 3 shows complementary workflows: MALDI-MSI uses a tissue-section branch, not an obligatory sequence downstream of bulk extraction and HPLC.

Validation is method- and decision-specific. For quantitative HPLC assays, report selectivity, calibration model and range, accuracy, repeatability and intermediate precision, recovery, detection/quantification limits where appropriate, carryover, solution stability, and robustness in each matrix; distinguish native analytes from hydrolysed totals. The cited QAMS work reports satisfactory linearity, precision, accuracy, and agreement with external-standard quantification, whereas the HPLC–DAD/NIR study reports explicit precision, 24 h stability, reproducibility, and external prediction rates [37,58]. Fingerprints require repeatability, intermediate precision, and robustness; QAMS correction factors need cross-column/laboratory confirmation; NIR and DNA/origin models need independent grouped external validation. LC–MS profiling studies [21,22] support confidence-graded identification but should not be described as fully validated quantitative release assays unless their quantitative validation is separately demonstrated. MALDI-MSI requires reproducible tissue preparation and calibrated orthogonal confirmation before absolute-concentration claims.

### 4.2. HPLC Coupled with Electrospray Ionization Tandem Mass Spectrometry (HPLC-ESI-MS/MS)

Jin et al. integrated chromatographic retention, UV and negative-ion mass spectra, reporting 41 constituents as identified or tentatively characterized [21]. The analytical confidence is compound-specific. A match to an authentic standard under comparable conditions is stronger than a database match or a neutral-loss pattern, and MS/MS alone may not distinguish positional glycosides.

UPLC/Q-TOF-MS provides complementary accurate-mass information; the 30 assignments in Gao et al. were explicitly tentative and informed by seven standards [22]. “First reported from the species” in an annotation study is not equivalent to the isolation and complete elucidation of a new compound. Novel structures or uncertain substitution patterns should ideally be confirmed after isolation by orthogonal spectroscopy, particularly NMR.

A later study compared water and ethanol extracts and reported 101 parent-related or metabolite assignments in rat serum, comprising 15 prototype compounds and 86 metabolites [50]. These are circulating-profile assignments, not 101 newly identified constituents of the plant. The study adds exposure-related evidence but does not provide a comprehensive human pharmacokinetic description of the review’s proposed marker classes.

### 4.3. Spatial Metabolomics by MALDI-MSI

MALDI-MSI preserves spatial information that is lost during homogenization. Wang et al. combined root imaging, UPLC-ESI-MS/MS and transcriptomics across three developmental stages [6]. The accessible abstract does not support a universal developmental trajectory or a tissue-specific stilbene assignment.

**Figure 4:**
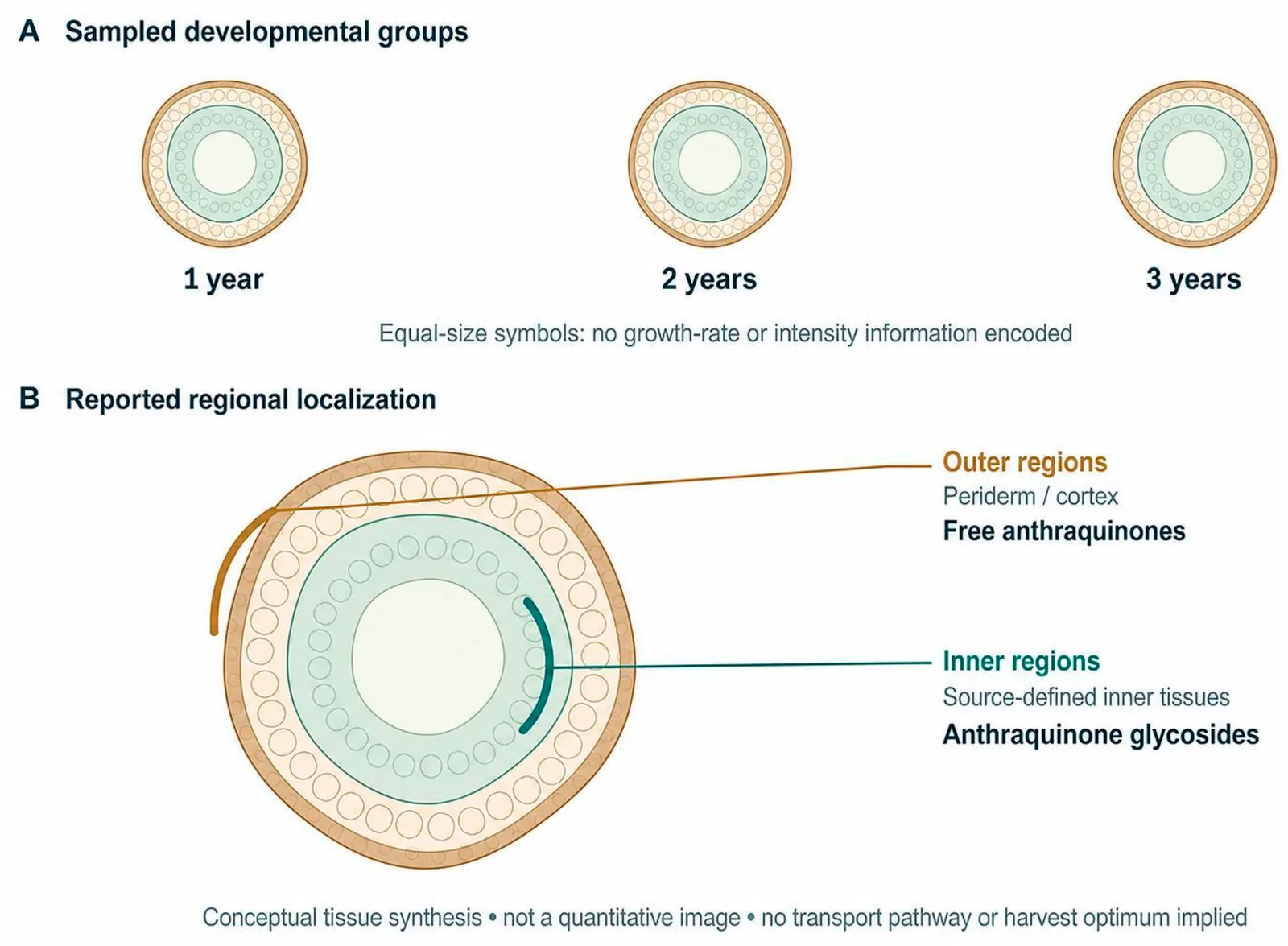
Qualitative tissue and developmental synthesis based on Zhao et al. [23]. (**A**) One-, two-, and three-year-old roots are represented by equal-size section symbols without an abundance or growth scale. (**B**) The source reports preferential free-anthraquinone localization in outer periderm/cortical regions and glycoside enrichment in inner regions. The source uses vascular-cylinder/pith terminology; this coarse illustration does not resolve phloem from xylem or establish identical root/rhizome anatomy. Cell boundaries and positions are conceptual, not reconstructed histology. The source combined imaging with targeted measurement of 11 anthraquinones. No concentration scale, monotonic age trend, transport direction, or optimum harvest age is inferred.

Zhao et al. examined one-, two- and three-year-old *R. tanguticum* roots and reported preferential free-AQ signals in periderm/cortical regions and glycoside enrichment in inner regions termed the vascular cylinder and pith by the authors, alongside targeted measurements of 11 AQs [23]. Figure 4 summarizes only this qualitative regional distinction. Anatomical region names are retained at the source’s level of resolution; the schematic does not establish exact phloem/xylem boundaries or imply identical root and rhizome anatomy.

Ion images depend on matrix application, section preparation, ionization efficiency, local suppression, adduct chemistry and normalization. Therefore, brighter pixels do not automatically mean higher absolute concentration across chemically different analytes. Orthogonal histology, accurate mass, fragmentation and reference standards strengthen spatial assignments, while calibrated tissue extraction can provide complementary quantitative evidence. Co-localization of transcripts and metabolites does not prove biosynthetic flux, transport direction or causation. Functional perturbation and isotope tracing would be needed to distinguish these possibilities.

### 4.4. Statistical Optimization and Experimental Design

RSM and orthogonal designs address planned experimental factors; chemometric discrimination addresses sample patterns and prediction. These purposes should not be conflated. An extraction optimum applies to the tested material, response and factor ranges, and should be confirmed with independent experiments. For multiple responses, normalization and weighting must be justified because maximizing crude yield may oppose preservation of a labile target.

The rhubarb DES study illustrates integration of RSM with ANN–GA modeling [54]. Complexity does not replace validation: training/test separation, overfitting control and experimental confirmation remain necessary. Similarly, predictive models of ecological quality in *R. tanguticum* have been combined with cultivation verification [61], but their predictions concern the sampled environmental domain, not universal compositional limits.

### 4.5. Chemometric Evaluation by Gray Relational Analysis (GRA)

GRA ranks samples relative to a chosen reference sequence, not an independently established biological optimum. Treating the maximum of every constituent as “best quality” is unjustified when constituents may contribute differently to activity and toxicity.

A more recent species-specific HPLC/NIR investigation included GRA in its spectrum–effect analysis [58]. Such association scores prioritize candidates but do not prove constituent synergy or causal contribution.

Species-specific work has applied PCA-based multivariate analysis to anthraquinones, dianthrones and tannins [27]. Stable-isotope and elemental measurements combined with chemometrics have addressed geographical origin using 190 *R. tanguticum* samples from 38 locations [60]. NIR models have predicted chemical antioxidant-assay responses when calibrated against chromatographic and assay data [58]. Origin classification and prediction of an in vitro antioxidant endpoint are distinct achievements, neither of which validates therapeutic superiority.

Validation should separate samples by meaningful units such as site, harvest or batch to limit leakage. Preprocessing, feature selection and model tuning must be confined to training data. Independent populations, external seasons and instruments provide stronger tests of transferability than random splitting of closely related specimens. Accuracy estimates, uncertainty and the intended decision threshold should be reported without presenting exploratory clustering as a validated authentication test.

## 5. Structure–Activity Relationships of Anthraquinones

The A–D provenance categories described in Section 1.4 are particularly important for structure–activity interpretation. Studies reporting *R. tanguticum* extracts or fractions are species-relevant but do not automatically establish authenticated category A evidence or matched-series SAR [12,13]. Species-derived isolation evidence (category B) can establish chemical occurrence without identifying the active principle of a whole extract [25,28]. *R. officinale* fermentation and glycosyltransferase studies provide category C comparisons [45,46]. Broader emodin reviews and anthraquinone-series assays are category D evidence, informative for hypotheses but not species-specific extract efficacy [38,39,62].

Nam et al. compared anthraquinone and hydroxy-substituted derivatives in chemical assays and RAW 264.7 cells, supporting assay-specific relationships between hydroxylation patterns and antioxidant/inflammatory endpoints [62]. This does not establish a universal potency order for the five major *R. tanguticum* aglycones. In particular, the rhein carboxyl group does not, by itself, prove nephroprotective specificity, nor does the aloe-emodin hydroxymethyl group establish superior antitumour efficacy. Solubility, ionization, permeability, metabolism, protein binding and assay conditions can all affect observed responses.

Substitution-based SAR claims require consistent atom numbering and matched experimental comparisons. Glycosylation may alter exposure and microbial conversion, but neither reduced direct activity nor increased aqueous solubility should be asserted without compound- and condition-specific data.

### 5.1. Anthraquinone–Tannin Pharmacological Interaction

A preparation-dependent balance between laxative constituents and astringent polyphenols is a useful hypothesis in the broader rhubarb literature [2]. It is not a demonstrated quantitative interaction model for *R. tanguticum*. Claims of synergy or antagonism require controlled combinations, concentration–response data and an appropriate additivity model. Correlations between peaks and assays cannot establish synergy.

Processing-induced conversion of glycosides to aglycones has direct species evidence under scorch-treatment conditions [56]. This does not establish a general mapping from decoction duration or dose to purgative versus astringent effects. Intact tannins also undergo chemical changes; they should not be designated universally heat-stable.

### 5.2. Pharmacokinetics, Toxicity and Safety Relevance

Extraction-dependent rat circulating profiles provide a useful starting point for multi-component exposure studies [50]. Priorities include authentic-standard confirmation, time-resolved concentration data, parent/metabolite distinction, dose dependence, oral bioavailability and interactions with gut microbiota. In vitro fermentation is complementary but cannot establish systemic exposure [34,46].

The emodin literature and broader rhubarb reviews describe both beneficial preclinical endpoints and potential toxicity, including hepatic concerns [38,63]. Comparative rhubarb research has combined chemometrics with zebrafish acute-toxicity testing, proposing candidate toxicity-associated constituents [64]. Such candidates are not validated human safety markers, and molecular docking does not demonstrate target engagement. Safety cannot be inferred from botanical identity or high marker concentration alone.

The EMA’s safety restrictions concern the *R. palmatum*/*R. officinale* preparations within its scope, including limits on duration of use and contraindications [9]. They provide relevant caution but should not be represented as a directly validated *R. tanguticum* dosing standard. Experimental benefits of isolated polysaccharides in rodent colitis do not justify recommending anthranoid-containing whole rhubarb to patients with inflammatory bowel disease.

## 6. Proposed Quality Control Framework

### 6.1. Current Official Controls and Limitations

The 2025 edition of the Pharmacopeia of the People’s Republic of China was promulgated by the National Medical Products Administration and National Health Commission and took effect on 1 October 2025 [65]. The Dahuang (Rhei Radix et Rhizoma) monograph accepts the dried roots and rhizomes of *R. palmatum* L., *R. tanguticum* Maxim. ex Balf., and *R. officinale* Baill. interchangeably under the Dahuang designation. Official identity controls include macroscopic and microscopic examination and TLC comparison with reference crude drug and reference standards. TLC purity/adulteration screening checks for unauthorized Rheum and rhaponticin-associated fluorescence under ultraviolet light at 365 nm. General quality tests include moisture/loss on drying, total ash, acid-insoluble ash, and water-soluble extractives. Quantitative control uses dual HPLC assays for acid-hydrolyzed total anthraquinones and unhydrolyzed free anthraquinones, the latter measured as the sum of aloe-emodin, rhein, emodin, chrysophanol, and physcion. The monograph also contains preparation-specific provisions for processed decoction pieces, including wine-processed, steamed/prepared, and charred rhubarb [65].

Because these three botanical sources are accepted interchangeably under the Dahuang designation, the official source definition and drug-level tests do not distinguish *R. tanguticum* from *R. palmatum* or *R. officinale*. The publicly accessible EMA material concerns specified preparations of *R. palmatum* and *R. officinale* (or hybrids), not a species-specific authorization for *R. tanguticum* [9]. This review proposes voucher-linked morphology with validated DNA/Bar-HRM for species discrimination; direct measurement of intact anthraquinone glycosides and sennosides A/B; reconciliation of conflicting stilbene, tannin, phenylbutanone, and polysaccharide evidence; and matrix-specific validation from raw material through processed pieces, extracts, and finished products. These are research extensions, not current official requirements or validated release specifications, and they do not replace applicable Chinese Pharmacopeia controls.

### 6.2. Proposed Multi-Class Quality Assessment Panel

Candidate markers are prioritized against six qualitative criteria: botanical specificity; abundance and between-batch reproducibility in authenticated material; chemical stability and sensitivity to drying/processing; analytical selectivity and validation feasibility; availability of reference standards; and relevance to the intended product’s composition, biological performance, or safety. Identity markers and composition markers are treated as separate roles. Because representative authenticated-batch data are insufficient for calibrated weights or pass/fail scores, the framework is qualitative and matrix-specific.

Section 6.1 describes the official baseline—interchangeable Dahuang source definition; macroscopic/microscopic and TLC identity/adulteration tests; general quality tests; and dual HPLC anthraquinone assays. The Chinese Pharmacopeia’s free-anthraquinone sum provides a shared compositional baseline, not species identity. Table 9 therefore separates botanical identity from composition monitoring and prioritizes species-level discrimination and direct native glycoside/sennoside assays as research extensions. Stilbene, tannin, phenylbutanone, and polysaccharide profiles remain candidates to reconcile and validate for their intended matrices; these extensions are not current official release requirements.

Selection should be driven by incremental information, analytical feasibility and the intended product rather than a predetermined marker count. A candidate can be informative for consistency without having established clinical efficacy. Conversely, a compound with the broad pharmacological literature may be unsuitable as an authentication marker if shared among species. Threshold derivation requires representative authenticated batches, validated analytical uncertainty, stability data and externally confirmed decision performance.

### 6.3. Matrix-Specific Implementation

Raw medicinal material, decoction pieces, extracts, and standardized finished products differ in intended use, processing history, and analytical matrix; they therefore should not share an unmodified release panel. Table 10 distinguishes current official controls from proposed research additions.

### 6.4. Proposed Quality-Control Workflow

**Figure 5:**
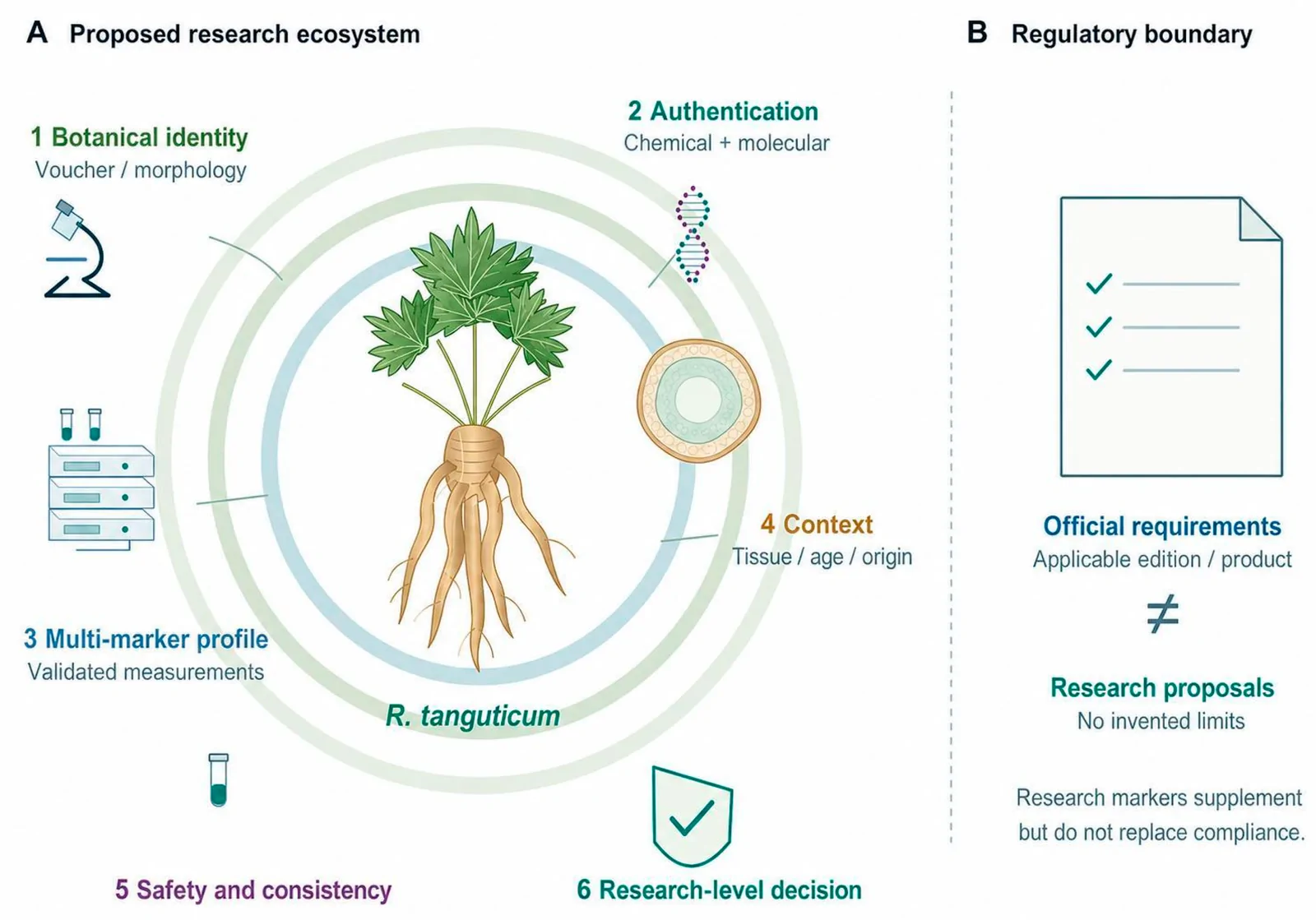
Proposed multidimensional quality-assessment ecosystem for R. tanguticum. (**A**) Proposed research ecosystem: six research dimensions address botanical identity, complementary authentication, multi-marker composition, tissue/developmental/environmental context, safety and consistency, and an independently validated decision process. Open rings organize overlapping research domains without quantitative weighting. (**B**) Regulatory boundary: the official requirements document is distinguished from the proposal: research markers cannot replace applicable regulatory requirements. No marker minima, universal similarity cutoff, or validated release algorithm is established [3,9,37,51,56,57,59,60,65,66].

Figure 5 proposes an evidence-tiered workflow, not an official standard. The first tier is identity: voucher-supported macroscopic and microscopic examination, documented supply-chain origin and appropriate molecular confirmation. Bar-HRM has been studied in rhubarb species and commercial preparations and can complement chemical testing, subject to DNA quality and the assay’s validated discrimination range [59]. Shared anthraquinone TLC spots alone are insufficient for species-level proof.

The second tier is chemical characterization using a reproducible fingerprint and validated multi-marker measurements. Free and conjugated analytes should be distinguished, and processed products evaluated in their own validated domain [66,67]. Spatial heterogeneity justifies representative sampling; MALDI-MSI remains a research tool rather than a routine release requirement [6,23].

The third tier contextualizes variation. Geographical-origin models [60], morphological-type comparisons [15], population genetics [3] and cultivation-validated ecological models [61] identify factors that can influence interpretation. Microbiome–metabolite studies add observational associations that should not be converted into causal quality rules [68]. Root age, drying and processing must be recorded alongside geography and genotype [51,56].

The fourth tier addresses safety, batch consistency and the decision rule. Applicable monograph and product-specific requirements must be met; additional research markers do not replace contaminant assessment or official identity tests. Statistical outliers should trigger investigation, including possible substitution, processing differences or analytical failure. Any proposed fingerprint or marker cutoff needs independent validation before use to accept or reject a batch. Higher constituent concentrations do not necessarily indicate greater safety or therapeutic benefit.

## 7. Conclusions and Future Perspectives

### 7.1. Summary

*R. tanguticum* has a chemically diverse evidence base spanning anthraquinones, dianthrones, stilbenes, tannin-related compounds, polysaccharides and minor constituents. Combining isolated structures and tentative annotations into a single count would overstate the established chemical inventory. Study-specific profiling counts should retain their identification confidence and sampling scope [21,22,23].

Extraction and preparative separation answer different questions. Solvent, thermal history and fractionation change the material ultimately analyzed or tested; reported HSCCC purities are not raw-plant extraction yields [28,31,50]. HPLC, LC-MS/MS and MALDI-MSI provide complementary compositional, structural and spatial information, but none independently establishes botanical identity, therapeutic benefit or validated product quality.

### 7.2. Research Gaps and Practical Solutions

First, establish a voucher-linked reference collection of *R. tanguticum* covering relevant populations, organs, developmental stages, harvests, and processing states. Deposit vouchers and record locality, organ, age, drying, and storage; combine morphology/microscopy with a validated DNA method where the matrix permits. This would make category-A evidence and species-discriminatory performance testable.

Second, resolve contradictory stilbene reports and tentative minor-compound annotations by re-examining authenticated material with authentic standards, orthogonal high-resolution MS/MS, and isolation/NMR for unresolved structures. Publish chromatograms, spectra, stable identifiers, and confidence levels; do not use unresolved rhapontin signals as universal presence/absence markers.

Third, compare extraction methods head-to-head on matched, authenticated lots with prespecified endpoints. Report dried crude-extract yield, analyte recovery per gram of starting material, parent/product mass balance, hydrolysis basis, solvent and energy use, and stability separately. Confirm model optima experimentally and include processing-specific root/rhizome material rather than transferring stem or other-species conditions.

Fourth, validate assays and prediction models for their intended decisions. Targeted chromatography requires matrix-specific selectivity, calibration/linearity, accuracy, precision, recovery, detection/quantification limits where relevant, solution stability, and robustness. QAMS correction factors, NIR models, DNA classifiers, and origin models require external testing across independent batches, sites, seasons, laboratories, and instruments; spatial imaging needs calibrated orthogonal confirmation before quantitative claims.

Fifth, develop separate research specifications for raw material, each decoction-piece type, extracts, and finished standardized products, while retaining the applicable official monograph as the regulatory floor. Add multi-component exposure, repeated-dose toxicology, processing-related transformation, and clinically relevant safety assessment before proposing biological release markers or product claims.

### 7.3. Concluding Remarks

The review supports three conclusions. First, the evidence for *R. tanguticum* is chemically diverse but uneven in authentication and structural certainty; species-reported findings should not be promoted to authenticated evidence without vouchers or equivalent checks. Second, extraction yield, marker recovery, chromatographic purity, and biological response are different endpoints, and analytical methods must be validated for their intended matrix and decision. Third, the 2025 Chinese Pharmacopeia Dahuang monograph specifies a shared multi-source drug through botanical-source definition, macroscopic/microscopic/TLC identity and adulteration controls, general quality tests, dual HPLC anthraquinone assays, and provisions for processed pieces [65]. Because the three source species are accepted interchangeably, these controls do not establish *R. tanguticum* alone. Species-specific authentication, intact glycoside/sennoside quantitation, reconciliation of secondary-class evidence, and matrix-specific validation are proposed research extensions. Reliable standardization therefore requires a traceable chain from authenticated material through processing, validated measurement, and safety interpretation; no single marker or higher concentration alone establishes species identity, therapeutic value, or safety.

## Figures and Tables

**Table 1:** Evidence-oriented summary of chemical classes reported for *R. tanguticum* and their provenance.

Chemical Class	Representative Evidence	Evidence Category/Provenance	Interpretation and Limitation	Sources
Anthraquinone aglycones	Emodin, rhein, chrysophanol, aloe-emodin, physcion	Species-reported analytical profiling [21,22]; authentication not independently verified (A not confirmed).	Major compositional targets; shared among official *Rheum* sources, so presence is not species identity or clinical efficacy.	[21,22]
Anthraquinone glycosides	Isolated emodin, aloe-emodin, chrysophanol, and physcion glucosides	B: compounds isolated from material reported as *R. tanguticum*; voucher authentication not independently audited.	Positional isomers require appropriate structural evidence; processing may change native conjugates.	[28,29]
Dianthrone glycosides	Sennosides A and B measured in variation types	Species-reported measurement [15,27]; authentication for A not independently verified. Metabolism context: D [30].	Metabolic activation is established in the broader anthranoid literature, not unique to this species.	[15,27,30]
Stilbenes	Resveratroloside; reports of rhapontin-related compounds	B: species-derived isolation reports [25,31]; C: comparative authentication evidence [32].	Conflicting reports require voucher-, structure-, and method-level reconciliation before diagnostic use.	[25,31,32]
Tannins and related phenolics	Tannin annotations, catechin, galloyl-related constituents	Species-reported profiles/isolation [21,25]; C comparative quantitative methods [4,33].	Gallic acid and catechin are not equivalent to intact tannin content; define the measured pool.	[4,21,25,33]
Polysaccharides	RTP/RTP1 study-defined preparations	Species-reported preparations [10,11,34]; independent authentication not established here.	Fraction names do not define equivalent molecular structures or purity.	[10,11,34]
Phenylbutanones	Glycosylated phenylbutanones; cis-p-coumaroylated derivative	B for an explicitly isolated derivative [35]; species-reported profiling [21].	Isolation evidence exists; a complete species inventory and routine marker value are unresolved.	[21,35]
Naphthalene derivatives and chromones	Reported within the broader rhubarb literature; tentative species annotations	C/D for the contextual literature [2]; annotation-level evidence in [22].	Species assignment and annotation confidence must be retained; no verified total is proposed.	[2,22]
Flavonoids and other constituents	Organ-resolved metabolomics and seed studies	Species-reported tissue studies [14,23,36]; authentication not independently verified for category A.	Organ-specific occurrence; untargeted assignments need confirmation and should not be generalized to roots.	[14,23,36]

**Table 2:** Principal free anthraquinones: chemical identity and analytical distinction [24].

Compound	Neutral Molecular Formula	Average Molar Mass (g mol^−1^)	Analytical Distinction
Emodin	C15H10O5	270.24	Constitutional isomer of aloe-emodin
Rhein	C15H8O6	284.22	Carboxyl-containing anthraquinone
Chrysophanol	C15H10O4	254.24	Methyl-substituted dihydroxyanthraquinone
Aloe-emodin	C15H10O5	270.24	Hydroxymethyl-substituted anthraquinone
Physcion	C16H12O5	284.26	Methoxy-containing anthraquinone

**Table 3:** Representative conjugated anthraquinones supported by species-derived preparative studies.

Parent Aglycone	Compound	Evidence/Method	Category/Provenance	Source
Aloe-emodin	Aloe-emodin 8-O-β-D-glucoside	Resin enrichment and HSCCC isolation	B; species-derived isolate; voucher not independently audited.	[28]
Emodin	Emodin 1-O-β-D-glucoside	Resin enrichment and HSCCC isolation	B; species-derived isolate; voucher not independently audited.	[28]
Emodin	Emodin 8-O-β-D-glucoside	Resin enrichment and HSCCC isolation	B; species-derived isolate; voucher not independently audited.	[28]
Chrysophanol	Chrysophanol 1-O-β-D-glucoside	Preparative HPLC/HSCCC	B; species-derived isolate; voucher not independently audited.	[29]
Chrysophanol	Chrysophanol 8-O-β-D-glucoside	Preparative HPLC/HSCCC	B; species-derived isolate; voucher not independently audited.	[29]
Physcion	Physcion 8-O-β-D-glucoside	Preparative HPLC/HSCCC; spelling normalized	B; species-derived isolate; voucher not independently audited.	[29]

**Table 4:** Sennoside evidence, provenance, and interpretation.

Analyte/Process	Evidence Scope	Evidence Category/Provenance	Appropriate Conclusion	Source
Sennoside A	*R. tanguticum* chemical measurement	Species-reported; authentication for A not independently verified.	Candidate additional compositional marker; process- and intended-use dependent.	[15,27]
Sennoside B	*R. tanguticum* chemical measurement	Species-reported; authentication for A not independently verified.	Candidate additional compositional marker; process- and intended-use dependent.	[15,27]
Microbial conversion of sennosides	The broader sennoside metabolism literature	D: compound-level metabolism evidence.	Deglycosylation and reduction contribute to rheinanthrone formation.	[30]
Fermentation of *R. officinale* extracts	Other-species in vitro model	C: other *Rheum* species.	Supports microbiota-dependent transformation, not human pharmacokinetics of *R. tanguticum*.	[46]

**Table 5:** Representative reported stilbenes with explicit evidence categories and authentication cautions.

Compound as Reported	Evidence/Method	Category/Provenance	Interpretation	Source
Resveratroloside	Preparative HSCCC with spectroscopic characterization	B: species-derived isolate [25]; voucher not independently audited.	Direct species-reported isolation; not a universal authentication marker.	[25]
trans-Rhapontin	HSCCC and NMR	B report [31]; C comparative conflict [32].	Reported isolation conflicts with some of the authentication literature; reconcile source and structure.	[31,32]
cis-Rhapontin	HSCCC and NMR	B report [31]; C comparative conflict [32].	Preserve stereochemical designation; same authentication caution.	[31,32]
trans-Desoxyrhaponticin	HSCCC and NMR	B: species-derived isolate report [31].	Do not substitute piceid or resveratroloside for this name.	[31]
Piceatannol 4′-O-β-D-(6″-O-gallate)-glucoside	Resin enrichment and HSCCC	B: species-derived isolation report [28].	Galloylated stilbene glycoside; source authentication remains to be independently checked.	[28]
Rhapontigenin and desoxyrhapontigenin	Bioactivity-guided fractionation	Species-derived fractions [12]; isolate provenance is not a clinical-efficacy claim.	Plant-pathogen assay context, not clinical antimicrobial efficacy.	[12]

**Table 6:** Reported HSCCC conditions and products in the stilbene-glycoside study [31].

Parameter	Reported Value	Interpretation/Evidence Scope
Biphasic solvent system	Chloroform:n-butanol:methanol:water, 4:1:3:2 (*v*/*v*/*v*/*v*)	Species-derived preparative isolation report (B); voucher authentication not independently audited.
Stationary/mobile phase	Upper phase/lower phase	Phase labels retained as reported; avoid assuming phase composition.
Flow rate	1.8 mL min^−1^	One reported separation condition.
Rotation and temperature	800 rpm; 25 °C	One reported separation condition.
Monitoring	UV at 280 nm	Method-monitoring condition.
Loaded sample	80 mg crude sample	Crude-load mass, not plant dry-weight mass.
trans-Rhapontin	25.5 mg; reported purity 99.6%	Purified mass and purity; not root extraction yield.
cis-Rhapontin	16.0 mg; reported purity 97.2%	Purified mass and purity; not root extraction yield.
trans-Desoxyrhaponticin	20.5 mg; reported purity 99.2%	Purified mass and purity; not root extraction yield.
Structural evidence	1H- and 13C-NMR; HPLC analysis	Reported structural support; botanical-source authentication remains a separate question.

**Table 7:** Reported extraction yields or recovery outcomes and method-specific strengths and limitations. Values are not directly comparable across different materials or denominators.

Method	Reported Yield/Recovery	Advantages	Limitations/Interpretation	Evidence Provenance
Aqueous reflux (the literature comparator)	0.672% total-anthraquinone assay yield (100 °C, 20 min, 1:15 solid:liquid, three extractions), as compiled in [55].	Simple apparatus and familiar water-based process.	This is a value compiled from a separate report, not a same-lot comparison with SWE; botanical identity and assay comparability were not independently checked here. It is target-analyte yield, not crude extract mass.	C/comparator summarized in [55]; not independently authenticated as *R. tanguticum*.
Petroleum-ether reflux (the literature comparator)	1.121% total-anthraquinone assay yield (95% petroleum ether, 60 °C, 30 min, 1:5, three extractions), as compiled in [55].	Nonpolar solvents can recover lipophilic constituents.	Separate study and solvent system; solvent hazard, recovery, residues, and plant-source comparability require evaluation. Not a crude-extract yield.	C/comparator summarized in [55]; taxon/material not verified here.
Ultrasound-assisted extraction (UAE)	The comparison table in [55] reports 1.755%; its discussion text reports 1.775%. The cited UAE study used *R. palmatum*, 84% methanol, 67 °C, 33 min, 1:15, 250 W [55].	Cavitation can enhance mass transfer and shorten extraction time.	Internal discrepancy in [55] (1.755 vs. 1.775); confirm against the cited primary paper before quantitative reuse. Different species and solvent mean it is not a head-to-head *R. tanguticum* result.	C/other-species comparator; not direct *R. tanguticum* evidence.
Microwave-assisted dynamic extraction (MAE)	1.970% total-anthraquinone assay yield, as summarized in [55] (10 min, 520 W, 4 mL min^−1^; solvent reported as 60% petroleum ether).	Rapid volumetric heating and dynamic solvent contact may reduce processing time.	Value is from a separate literature method; species and analytical basis are not independently verified. Hot spots, pressure control, scale-up, and thermal transformation need assessment.	C/comparator summarized in [55]; not a same-lot comparison.
Supercritical-fluid extraction (SFE-CO2)	2.630% total-anthraquinone assay yield after 60 min at 18 MPa and 60 °C with 450 mL of 95% petroleum-ether entrainer, as compiled in [55].	CO2 can be recovered and the process can limit conventional organic-solvent use when operated without large entrainer loads.	The reported entrainer volume and separate source limit a “green” inference; pressure, solvent recovery, and matrix comparability matter. The cited comparison concerns *R. palmatum*.	C/other-species comparator summarized in [55].
Subcritical-water extraction (SWE)	3.581 ± 0.246% (*n* = 3) total-anthraquinone assay yield at 54 min, 170 °C, 2.0 mL min^−1^ and 5 MPa [55]. Ten grams of dried *R. tanguticum* root were extracted; total anthraquinones were measured spectrophotometrically against 1,8-dihydroxyanthraquinone and expressed relative to starting material.	Direct species-reported root study; water is the solvent, and an optimized condition was confirmed experimentally.	High temperature and pressure may transform constituents; the assay is a total colorimetric-equivalent measure, not LC-resolved recovery of each native compound. Equipment, energy, safety, and scale-up require evaluation.	Species-reported primary study; voucher authentication not independently verified (A status unresolved).
Aqueous/ethanolic extraction and stem-process optimization	The water/ethanol study reports comparative metabolomic and rat-serum profiles [50]. The stem-specific orthogonal-design study [52] reports an abstract-defined mean crude extract yield of 38.65% for *R. tanguticum* stems (12-fold water; three 1 h decoctions). This is a stem crude-extract yield, not a root/rhizome yield or a total-anthraquinone assay yield.	Useful for comparing extraction-dependent chemical profiles and exposure-related signals.	The 38.65% value is defined by the study abstract and applies to stems only; it cannot be transferred to medicinal roots/rhizomes or compared directly with target-analyte recovery. Interpret the response using the study’s own denominator and matrix.	Species-reported stem study [52]; not evidence of root/rhizome yield; authentication not independently verified.
Deep eutectic solvent (DES)	No comparable *R. tanguticum* crude- or target-yield value verified; the TBAB–polyol paper is treated as a *Rheum* methodological comparator [54].	Solvent polarity can be tuned and vapor pressure may be low.	Viscosity, toxicity, biodegradability, recovery, residual solvent, and product compatibility need process-level assessment; low volatility alone does not establish environmental superiority.	C/comparative *Rheum*-method evidence; not a confirmed *R. tanguticum* recipe.
Resin enrichment/HSCCC	Resin-elution analyte recoveries of 89.0%, 85.9%, 82.3%, and 84.9% for four targets [28]. These are recoveries after enrichment, not raw-plant extraction yields.	Selective enrichment; HSCCC avoids irreversible adsorption on a solid stationary phase and can furnish purified compounds.	Purification losses, solvent use, phase-system selection, throughput, and solvent handling; recovered purity is not batch composition.	B/species-derived isolation report; source authentication not independently verified.
Preparative HSCCC	An 80 mg crude load yielded 25.5, 16.0, and 20.5 mg of three stilbene glycosides, with reported purities of 99.6%, 97.2%, and 99.2% [31]; another study isolated 10.2 mg resveratroloside and 26.7 mg catechin from 250 mg crude [25].	Useful for structural elucidation and reference-material preparation; load and purity are reported.	Isolated masses from crude loads are not yields per gram of root and are not directly comparable with extraction yields.	B/species-derived isolation reports; authentication status unresolved.
SWE followed by HSCCC/preparative HPLC	From a 260 mg SWE crude-extract load, HSCCC yielded 13 mg and 26 mg of compounds I and II plus a 56 mg mixed fraction of III/IV; prep-HPLC then yielded 16 mg and 19 mg of III and IV. Reported purities were 93.6%, 97.9%, 98.9%, and 98.3%, respectively [55].	Shows an extraction-plus-purification route and produces structurally characterized reference compounds.	Isolated masses and purity describe downstream fractionation, not root extraction recovery or a batch specification.	Species-reported primary study; authentication status unresolved.

**Table 8:** Analytical capability, reported validation evidence, and remaining limits of cited methods.

Method and Evidence Provenance	Validation/Evidence Reported	Appropriate Use and Limitation
HPLC–DAD fingerprint [57]—species-reported; category A not independently confirmed	A species-reported fingerprint study assigned 21 characteristic peaks using retention and UV data and compared multi-sample fingerprints.	Supports profile comparison, not species identity or efficacy. The cited record does not establish a full set of quantitative linearity, recovery, and inter-laboratory transfer metrics.
Targeted HPLC/QAMS [33,37]—C: comparative multi-source *Rheum*	The multi-marker QAMS study reported satisfactory linearity, precision, and accuracy; QAMS results for eight anthraquinone glycosides were not significantly different from external-standard measurement across 28 batches representing three *Rheum* sources [37].	Strongest cited quantitative precedent, but not a species-exclusive *R. tanguticum* validation. Reference factors, selectivity, recovery, stability, and transfer across matrix, column, and laboratory still need confirmation.
LC–DAD–ESI-MS/MS [21]—species-reported; category A unverified	Forty-one constituents were identified or tentatively characterized using chromatographic, UV, and MS evidence; confidence differed by compound and standard support.	Useful for class profiling and structural hypotheses. It is not, by itself, a validated quantitative release assay; positional isomers require standards or orthogonal confirmation.
UPLC/Q-TOF-MS [22,50]—species-reported; category A unverified	The *R. tanguticum* profiling study reported 30 tentative assignments informed by seven standards [22]; the water/ethanol comparison separately described plant and rat-serum profiles [50].	Accurate mass and MS/MS support annotation but do not necessarily resolve isomers, stereochemistry, or plant constituent versus circulating metabolite.
HPLC fingerprint + NIR/chemometrics [58]—species-reported; category A unverified	The 2025 study reported HPLC peak RSDs for precision (retention time 0.07%, area 0.98%), 24 h stability (0.50%, 1.27%), and reproducibility (0.65%, 1.15%). NIR external prediction rates were 87.03% for ABTS, 83.88% for DPPH, and 88.30% for FRAP; RPD values were approximately 2.43–2.63.	Provides explicit repeatability/stability and calibration evidence for the studied antioxidant-assay domain. It predicts in vitro assay responses, not clinical efficacy; independent populations, seasons, instruments, and batches are needed before transfer.
MALDI-MSI [6,23]—species-reported; category A unverified	Reported spatial localization of assigned ions in root sections, complementary to bulk extraction and targeted measurement.	Spatial ion intensity is not automatically absolute concentration. Matrix effects, ion suppression, tissue preparation, normalization, and calibration limit routine quantitative interpretation.
Bar-HRM/stable isotopes and chemometrics [59,60]—comparative identity/origin evidence (C)	Applied to species discrimination or geographical-origin classification; the appropriate validation endpoints are specificity, sensitivity, classification error, and external-site performance rather than chromatographic linearity.	Identity and origin do not measure chemical potency. Independent populations, seasons, instruments, and blinded external test sets are needed to test transferability.

**Table 9:** Candidate research panel and qualitative prioritization by marker role and product matrix; not an official or validated release standard.

Candidate/Domain	Evidence Category	Why Prioritize It?	Best-Fit Matrix	Validation Gate/Limitation
Botanical identity (gate, not a chemical marker)	Official Chinese Pharmacopeia: shared Dahuang source definition and macroscopic/microscopic/TLC identity and adulteration controls [65]. Research extension: voucher-linked morphology/microscopy plus validated DNA/Bar-HRM for species-level discrimination [59].	Compendial source and identity controls support Dahuang compliance but accept the three sources interchangeably; species-level discrimination requires orthogonal, voucher-linked evidence.	Raw material: primary. Decoction pieces: use only with validated DNA suitability; processed extracts/finished products: DNA may be degraded, so retain supplier traceability and orthogonal chemistry.	Validate inclusivity/exclusivity, sensitivity to mixtures, and performance on processed matrices. Shared anthraquinones cannot substitute for species identity.
Five free anthraquinones: emodin, rhein, chrysophanol, aloe-emodin, physcion	Official Chinese Pharmacopeia: dual HPLC assays for acid-hydrolyzed total and unhydrolyzed free anthraquinones; the free assay sums five aglycones [65]. Species-reported analytical studies [21,33].	High analytical feasibility and reference-standard availability; useful shared Dahuang composition anchors and processing/safety context, but not species-specific.	Raw material, decoction pieces, extracts, and finished products when relevant to intended composition.	Validate free versus hydrolysis-defined total fractions, matrix recovery, stability, and measurement uncertainty. Do not use alone as an *R. tanguticum* authentication test.
Selected anthraquinone glycosides and sennosides A/B	B for isolated glycosides [28,29]; species-reported sennoside measurements [15,27] (A authentication not independently verified).	Adds native-conjugate coverage, processing sensitivity, and laxative/safety relevance; standards/isomer separation can be limiting.	Raw material and decoction pieces first; extend to extracts/finished products when processing or product claims make native forms relevant.	Resolve positional isomers; test hydrolysis/heat stability and recovery; do not infer a biological effect from concentration alone.
Resveratroloside and other confirmed stilbenes	B species-derived isolation reports [25,31]; comparative conflict [32].	Potentially useful orthogonal chemistry, but botanical specificity is unresolved and the rhapontin reports conflict.	Research authentication panel for raw material; consider processed products only after source reconciliation and matrix validation.	Resolve voucher, structure, nomenclature, and sensitivity differences; no universal diagnostic presence/absence rule proposed.
Tannin-related pool (defined intact tannin/procyanidin measures plus selected monomers)	Species-reported profiles/isolation [21,25] and comparative methods [4,33] (C where other-source data are used).	May capture a distinct compositional dimension; broad class assays are method- and calibration-dependent; gallic acid or catechin alone is not total tannin.	Raw material, decoction pieces, and water/alcohol extracts when the intended product warrants it.	Define the chemical pool, extraction, calibration equivalents, interference, and processing stability; avoid colorimetric totals as species identity.
Phenylbutanone representatives	B for a reported isolate [35]; species-reported profiling [21].	Potentially broadens chemical-class coverage; lower routine priority until abundance, between-lot reproducibility, and standards are established.	Optional profiling for raw material and research extracts; not a default release panel.	Confirm structure with standards/orthogonal spectra and demonstrate incremental discrimination or product relevance.
Polysaccharide fraction (product-specific)	Species-reported preparations [10,11,34]; fraction names are not structural identities.	Relevant when the marketed or experimental product is a polysaccharide fraction; no assumption that it is a single HPLC–DAD analyte.	Dedicated polysaccharide extracts or finished products carrying a polysaccharide claim; not a universal small-molecule marker for crude rhubarb.	Characterize molecular-weight distribution, monosaccharides, linkages, purity, protein/phenolic carryover, and endotoxin where mechanistic claims are made.

**Table 10:** Matrix-specific quality-control tiers: official controls versus proposed research additions.

Product Matrix	Current Official Scope/Control	Proposed Supplemental Assessment	Interpretation/Caveat
Raw medicinal material (root/rhizome)	The 2025 Chinese Pharmacopeia defines Dahuang as dried roots/rhizomes of *R. palmatum*, *R. tanguticum*, or *R. officinale* and requires macroscopic and microscopic identification plus TLC comparison with reference crude drug and standards; TLC purity/adulteration screening includes rhaponticin-associated fluorescence under ultraviolet light at 365 nm. General tests include moisture/loss on drying, total ash, acid-insoluble ash, and water-soluble extractives. Dual HPLC assays cover acid-hydrolyzed total and unhydrolyzed free anthraquinones (five-aglycone sum) [65].	Voucher-linked morphology/microscopy plus validated molecular confirmation where species discrimination is required; direct intact glycoside/sennoside assays; multi-class fingerprint; record organ, locality, harvest, drying, and storage; assess contaminants under applicable rules.	The three source species are accepted interchangeably under Dahuang; compendial compliance does not distinguish *R. tanguticum*. DNA/Bar-HRM and expanded class profiles are research additions.
Decoction pieces (raw, wine-processed, cooked, charcoal)	The 2025 Chinese Pharmacopeia includes preparation-specific provisions for wine-processed, steamed/prepared, and charred Dahuang decoction pieces [65].	Document processing inputs/time/temperature; compare intact glycosides and sennosides, free/total anthraquinones, and defined tannin metrics in matched material; develop piece-specific reference ranges.	Do not transfer raw-drug thresholds unchanged to processed pieces; measure processing-sensitive parent/product pairs.
Extracts (aqueous, hydroalcoholic, purified or enriched)	Raw-drug criteria should not be assumed to constitute validated extract-release specifications; check applicable official product requirements.	Specify starting material, drug-extract ratio, solvent, process conditions, dry extract yield, target-marker recovery, transformation/mass balance, residual solvents, fingerprint, and stability in the extract matrix.	A process-specific profile and recovery are more meaningful than a raw-herb extractive limit; validate each solvent/process and intended extract.
Standardized finished products	Applicable product-specific registration and pharmacopoeial requirements remain controlling; no *R. tanguticum*-specific universal limits are proposed here.	Validate assay and uniformity in the finished formulation; quantify markers per dose, confirm release/dissolution where relevant, stability, contaminants, and exposure/safety for the intended use.	Use a product-specific release specification supported by independent batches and intended-use data; research markers cannot replace official requirements.

## Data Availability

This review reports no new experimental data. The underlying studies are cited in the reference list.

## Abbreviations

ABTS, 2,2′-azino-bis(3-ethylbenzothiazoline-6-sulfonic acid) radical-cation assay; ANN, artificial neural network; AQ, anthraquinone; Bar-HRM, DNA barcoding with high-resolution melting; BBD, Box–Behnken design; DAD, diode-array detection; DES, deep eutectic solvent; DPPH, 2,2-diphenyl-1-picrylhydrazyl radical assay; DSS, dextran sulfate sodium; FRAP, ferric reducing antioxidant power; GA, genetic algorithm; GRA, gray relational analysis; HPLC, high-performance liquid chromatography; HRE, heat-reflux extraction; HSCCC, high-speed counter-current chromatography; IRMS, isotope-ratio mass spectrometry; LC–MS/MS, liquid chromatography–tandem mass spectrometry; MAE, microwave-assisted extraction; MALDI-MSI, matrix-assisted laser desorption/ionization mass spectrometry imaging; NIR, near-infrared; NMR, nuclear magnetic resonance; PCA, principal component analysis; PLS, partial least squares; QAMS, quantitative analysis of multiple components by a single marker; Q-TOF, quadrupole time-of-flight; RPD, ratio of performance to deviation; RSD, relative standard deviation; RSM, response surface methodology; SFE, supercritical-fluid extraction; SWE, subcritical-water extraction; TCM, traditional Chinese medicine; TLC, thin-layer chromatography; TNBS, trinitrobenzene sulfonic acid; UAE, ultrasound-assisted extraction; UGT, UDP-glycosyltransferase; UHPLC, ultra-high-performance liquid chromatography; UPLC, ultra-performance liquid chromatography.
